# Garage-Fabricated, Ultrasensitive Capacitive Humidity Sensor Based on Tissue Paper

**DOI:** 10.3390/s22207885

**Published:** 2022-10-17

**Authors:** Asad Ullah, Muhammad Hamza Zulfiqar, Muhammad Atif Khan, Muhammad Ali, Muhammad Zubair, Muhammad Qasim Mehmood, Yehia Massoud

**Affiliations:** Innovative Technologies Laboratories (ITL), King Abdullah University of Science and Technology (KAUST), Thuwal 23955, Saudi Arabia

**Keywords:** flexible electronics, humidity sensors, parallel-plate capacitive sensors, relative humidity

## Abstract

The role of humidity sensors in different industries and field applications, such as agriculture, food monitoring, biomedical equipment, heating, and ventilation, is well known. However, most commercially available humidity sensors are based on polymers or electronic materials that are not degradable and thus contribute to electronic waste. Here, we report a low-cost, flexible, easy-to-fabricate, and eco-friendly parallel-plate capacitive humidity sensor for field applications. The sensor is fabricated from copper tape and tissue paper, where copper tape is used to create the plates of the capacitor, and tissue paper is used as a dielectric sensing layer. Along with the low cost, the high sensitivity, better response and recovery times, stability, and repeatability make this sensor unique. The sensor was tested for relative humidity (RH), ranging from 40% to 99%, and the capacitance varied linearly with RH from 240 pF to 720 pF, as measured by an Arduino. The response time of the sensor is ~1.5 s, and the recovery time is ~2.2 s. The experiment was performed 4–5 times on the same sensor, and repeatable results were achieved with an accuracy of ±0.1%. Furthermore, the sensor exhibits a stable response when tested at different temperatures. Due to the above advantages, the presented sensor can find ready applications in different areas.

## 1. Introduction

With the advancement of automation and technology, there is an exponential increase in the demand for electronic devices and sensors. This leads to an increase in the production of electronic devices, such as sensors, smartphones, computers, and IoT devices. However, the increase in the production of electronic devices is also increasing electronic waste (e-waste). E-waste has severe environmental implications as it is not degradable and can end up in landfills or oceans. It is estimated that the e-waste produced in 2021 alone is 57.5 million metric tons, of which only 17% has been recycled [1,2,3,4,5]. Therefore, there is a dire need for green electronics based on materials that are bio-friendly and naturally degradable. On the other hand, the demand for printed, flexible, and foldable electronics is also rising. Printed electronics offer advantages, such as low-cost, easy processing, lightweight design, and wearability. The applications of flexible printed electronics include organic light-emitting diodes (OLEDs), touchpads, electronic papers, and organic photovoltaics (OPVs) [6,7,8,9,10,11,12]. Many printed sensors, such as gas sensors [13], pressure sensors [14], humidity sensors [15], temperature sensors [16], flexible touch sensors [17], health monitors [18], and implantable devices [19], have been reported in the literature. Paper-based flexible materials are often preferred for fabricating flexible electronics, as compared to polymer-based materials, due to their low cost, eco-friendly profiles, and easy processing [20].

Among the sensors, humidity- or moisture-based sensors have been extensively studied due to their importance in different industries and field applications, such as agriculture, food monitoring, biomedical equipment, heating, and ventilation [21,22,23,24]. These sensors are a type of transducer that monitors the concentration of water molecules in the surrounding environment. In the agriculture sector, the role of humidity sensors is extremely important as the timely monitoring of humidity in fields can significantly improve the yields of crops [25]. The figures of merit of a humidity sensor are sensitivity, response time, accuracy, repeatability, and stability [26,27,28]. The humidity sensors are mainly of two types: capacitive-based and resistive-based. The capacitive humidity sensor makes use of a capacitor consisting of a hygroscopic dielectric layer sandwiched between two plates. The absorption of moisture in the dielectric layer changes its dielectric permittivity [29]. The change in dielectric permittivity results in a change in capacitance. By measuring the change in capacitance, the humidity level can be established.

On the other hand, resistive humidity sensors make use of a change in resistance to establish the level of humidity. Capacitive humidity sensors are often preferred over resistive sensors, owing to several advantages. The basic structure in capacitive humidity sensors is of two types: interdigitated (IDE) and parallel-plate (PP) [30]. In PP capacitive humidity sensors, the upper electrode is porous, the lower electrode is flat, and a hygroscopic substrate is present in between. The dielectric layer is the sensing layer that absorbs water molecules through the upper, porous electrode [31,32]. In general, PP capacitive sensors have a higher sensitivity as compared to IDE capacitive sensors [31].

In this work, we have presented a PP humidity sensor based on eco-friendly materials, such as copper tape and tissue paper. The plates of the capacitor are made of copper tape, whereas tissue is used as the hygroscopic dielectric layer to absorb the moisture. The lower plate is a plane of copper tape, while the upper plate has pores to allow moisture to be absorbed by the tissue. The performance of tissue as a sensing layer is superior to various polymer-based sensing layers, such as polyethylene terephthalate (PET), polyimide (PI), or Kapton, in terms of sensitivity, response time, and stability. The sensor was tested for the range of 40% to 99% RH, and the capacitance varied linearly with RH from 240 pF to 820 pF. The sensor has a fast response and recovery times of 1.5 s and 2.2 s, respectively. The effect of temperature on the working of the sensor was also investigated, and the sensor exhibited reasonable sensitivity at different temperatures as well. The experiment was repeated 4–5 times on the same sensor, and the repeatability achieved was within ±0.1%.

## 2. Experiment

### 2.1. Sensor Fabrication and Characterization

**Figure 1:**
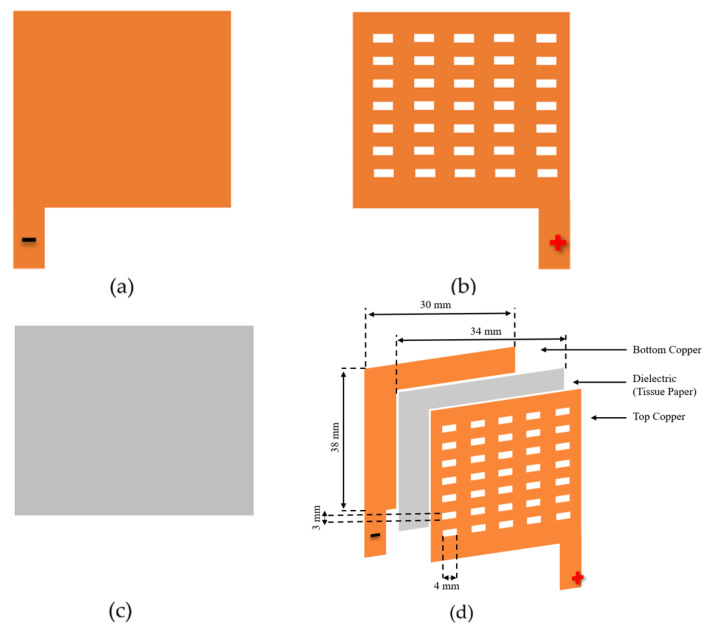
Copper-based, parallel-plate capacitive humidity sensor: (**a**) Lower side; (**b**) Upper side with meshes; (**c**) Tissue paper as a sensing layer; (**d**) Fabricated sensor.

Figure 1 illustrates the fabrication process of the copper-based, parallel-plate humidity sensor. The sensor works on the principle of change in capacitance with humidity. The plates of the capacitor are made from commercially available copper tape. One plate of the capacitor is composed of a plane of copper tape, while the other plate is composed of an array of meshes, as shown in Figure 1. A layer of tissue paper is placed in between the copper plates as a dielectric, as well as a sensing, layer. The reasons for using tissue paper as a sensing layer are its high clarity, biodegradability, low cost, and high sensitivity to humidity. On the other hand, copper has high electrical and thermal conductivity, thus making it a suitable material for the plates of capacitors.

**Figure 2:**
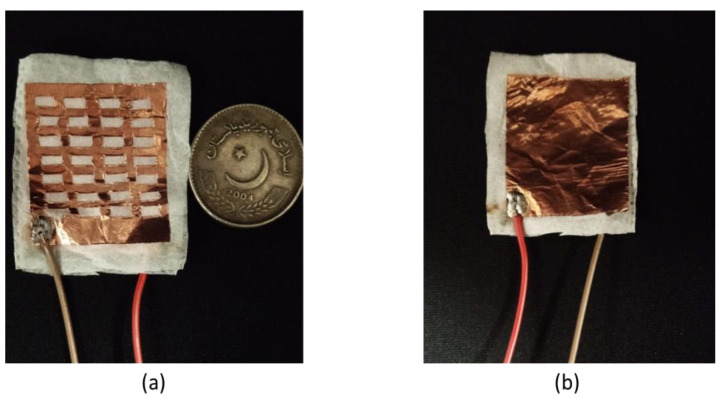
Fabricated parallel-plate sensor: (**a**) Front view of the sensor; (**b**) Back view of the sensor.

The physical dimensions and a picture of the sensor are shown in Figure 2. The size of each capacitor plate is 38 mm × 30 mm. The sensing layer is about 4 mm larger than the plates on all sides. The size of the sensing layer was kept large to ensure maximum electrical isolation between the plates. The size of each square on the upper plate is 4 mm × 3 mm. The thickness of the tissue paper is 0.3 mm, and that of the copper tape is 0.8 mm. These dimensions were optimized by making sensors of different sizes and shapes and then comparing their performance levels. It was observed that, in general, the sensitivity of the sensor increases with an increase in the size of the sensor. However, the presented dimensions were optimized for the highest performance. This sensor is cost-efficient, easy to fabricate, and does not require any specialized fabrication process.

**Figure 3:**
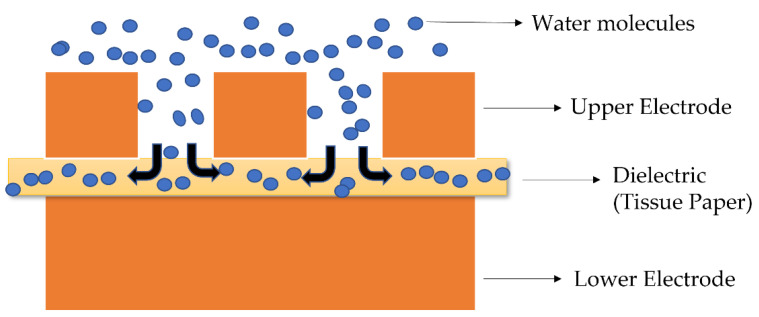
The working principle of a parallel-plate, capacitor-based sensor.

A capacitive sensor works on the principle of change in capacitance as the humidity varies [33,34,35,36,37]. This principle can be explained with the help of the diagram in Figure 3. One plate of the sensor is porous so that the water molecules in the air can diffuse through this array of meshes and reach the underlying tissue. The tissue paper is a good absorber and desorber of water as it has plenty of hydrophilic OH groups that can adsorb water molecules via hydrogen bonding. The water molecules are absorbed into the tissue and alter the dielectric constant of the tissue. This, in turn, changes the overall capacitance of the sensor, as capacitance strongly depends on the dielectric constant of the dielectric material. The higher the humidity, the higher the diffusion of water will be, and the higher the change in the capacitance will be. So, one can estimate the humidity from the change in the capacitance. Since water has a high dielectric constant of about 80, a slight change in the humidity can cause a measurable change in the capacitance. The high dielectric constant of water accounts for the high sensitivity of the sensor. The sensitivity can be further increased by increasing the area of the sensors as it will allow more water molecules to diffuse. However, a compromise must be made between the area of the sensor and the sensitivity. The presented sensor has several advantages, such as high stability, repeatability, linear behavior, recoverability, better response time, ease of fabrication, and cost efficiency.

### 2.2. Experimental Setup

**Figure 4:**
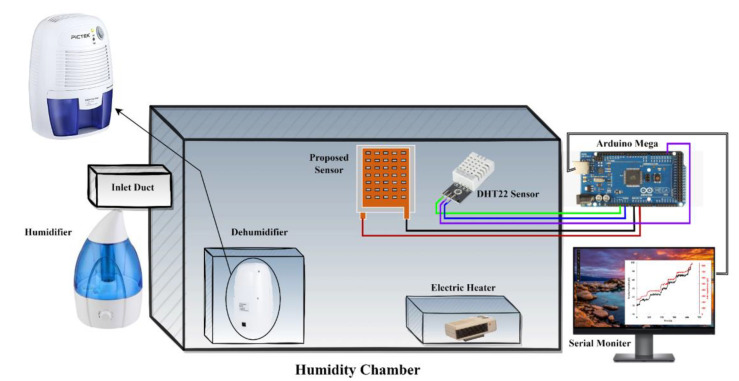
Experimental setup for measurement of humidity.

The sensor was tested in a homemade humidity chamber equipped with a heater, humidifier, and dehumidifier so as to control the humidity as well as the temperature as shown in Figure 4. A DHT22 interfaced with an Arduino Mega 2560 was used to measure the temperature and humidity inside the chamber. Using this setup, the chamber temperature can be varied between 25 °C to 40 °C, and the RH can be varied from 40% to 100%. Our capacitive humidity sensor was also placed in the chamber, and the changes in capacitance at different values of RH were measured using the Arduino Mega. The response of the sensor is observed through a serial monitor.

**Figure 5:**
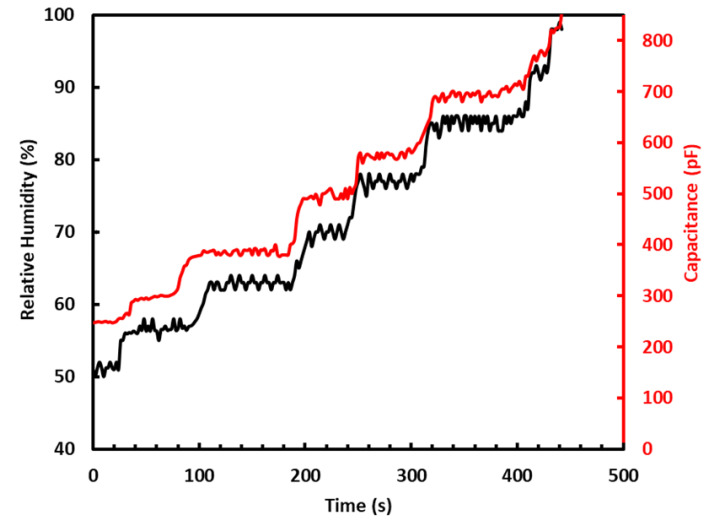
Change in %RH and capacitance of sensor as a function of time.

To test the sensor, the RH in the chamber was initially set to 45%. At this value of RH, the sensor had a capacitance of 240 pF, as measured by the Arduino. The RH was gradually increased to 100% by turning on the humidifier. The increase in RH increased the capacitance of the sensor, which reached 850 pF at 100% RH, as shown in Figure 5. During this measurement process, the temperature in the chamber was kept constant at 26 °C with the help of the heater and monitored using the DHT22. To reduce the level of RH in the chamber, the humidifier was turned off, and the dehumidifier was turned on. The sensor’s recovery time was measured by reducing the level of RH in the chamber, which will be discussed in a later section of this paper. It was observed that, as the RH was reduced in the chamber, the capacitance of the sensors also was reduced along with it. The measurements were repeated multiple times over weeks, and the sensor’s response remained unchanged. The measured results demonstrate that tissue can be a good absorber of water molecules and can induce sufficient change in the capacitance of the sensor. Moreover, the obtained results were repeatable and stable over a period of time. The sensor’s response was also measured at different temperatures, which will be discussed in detail in a later section of this paper.

## 3. Results and Discussion

**Figure 6:**
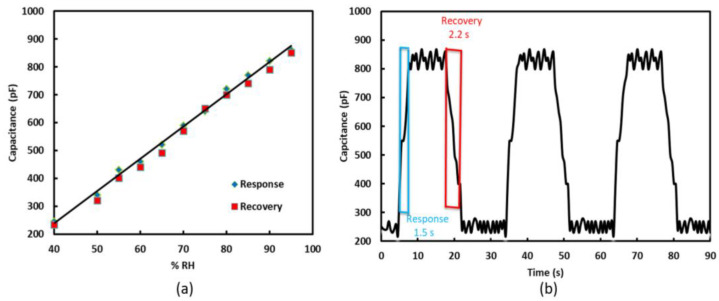
Response and recovery of sensor: (**a**) Relation between capacitance and RH; (**b**) Transient response of the sensor.

The sensor has a capacitance of 240 pF at 45% RH. When RH is increased with the help of the humidifier, the capacitance increases along with it. Different values, such as 60%, 70%, 80%, 90%, and 100%, of RH were maintained inside the chamber, and the capacitance values varied in accordance instantly. The measured values of the sensor’s capacitance at different levels of RH are shown in Figure 6a. A linear trend exists between capacitance and RH, which can be used to determine RH from the sensor’s capacitance. Further, the sensor’s capacitance at any value of RH remains essentially unchanged if the humidity is increased up to that point or reduced from a higher value to that point, as indicated by the response and recovery times shown in Figure 6a.

Figure 6b exhibits the transient response of the sensor, which was used to calculate the response and recovery times. Response time is defined as the time taken by the sensor to achieve 90% of total capacitance change during absorption, whereas recovery time is the time taken by the sensor to achieve 90% of total capacitance change during desorption [38,39,40]. Response and recovery times are figures of merit for any sensor and indicate how fast a sensor can operate. As indicated in Figure 6b, the response time of the sensor is ~1.5 s, and the recovery time is ~2.2 s. These values are far superior to previous studies on humidity sensors based on polymer sensing layers [41] and manifest the advantages of using tissue as a sensing layer. The transient response also confirms that our sensor has high sensitivity, as well as repeatable and stable results. Overall, the sensor has a sensitivity of 11 pF/%RH [42]. The sensitivity can be calculated using the following equations [42]:

(1) $$S=\left(\frac{\Delta \left(C\right)}{\Delta \left(\%\;RH\right)}\right)$$

(2) $$S=\left(\;\frac{C_{99RH}-C_{45RH}}{\%RH_{99}-\%RH_{45}}\right)$$

where $C_{45RH}$ is the capacitance of the sensor at 45% RH, as in our case, and $C_{99RH}\;$ is the capacitance measured when RH is 99%. $\Delta \left(\%\;RH\right)$ and $\Delta \left(C\right)$ indicate the change in RH and capacitance, respectively.

**Figure 7:**
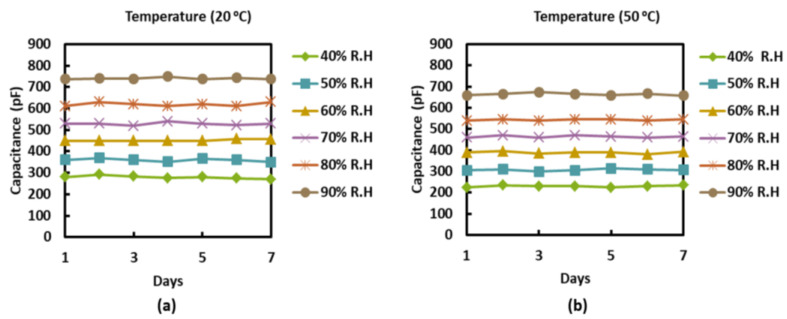
Capacitance of sensor measured at different values of %RH at (**a**) 20 °C and (**b**) 50 °C.

**Figure 8:**
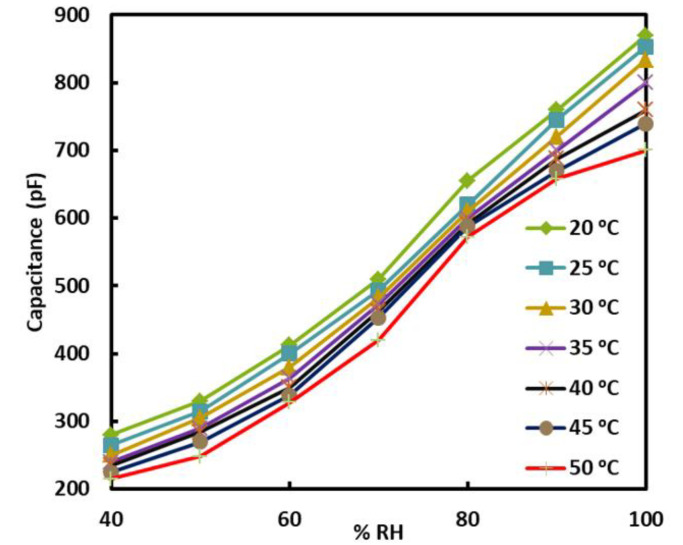
Capacitance of sensor versus %RH at inset temperatures.

The stability and repeatability of a sensor are key parameters for evaluating sensor performance. To test these, we performed repeated cycling of sensors at different temperatures for 7 consecutive days, as shown in Figure 7. The sensor exhibits repeatable and stable results, which manifest the use of the sensor for practical applications. In general, temperature also has a significant impact on the response of humidity sensors. This is primarily due to two reasons: first, due to changes in the conductivity of the materials used in the sensor [42,43,44], and second, due to the fact that the absolute humidity increases as the temperature is increased at a certain RH. We tested the sensor for temperatures ranging from 20 °C to 50 °C, as shown in Figure 8. In this figure, each curve shows the relation between the capacitance of the sensor and %RH at a particular temperature. A substantial change in the capacitance of the sensor can be observed as the temperature is varied. However, the sensitivity is unaffected by the change in temperature. To accurately monitor the RH using our sensor, one should follow the respective temperature curve at which the measurements are being performed. The presented results indicate that our sensor possesses several advantages, such as its low cost, stability, repeatability, and environmentally friendly design.

The comparison of the sensing parameters of our sensor, such as sensitivity, response time, and recovery time, with previously reported work is presented in Table 1. From the data presented in Table 1, it is clear that our proposed sensor exhibits remarkable results in terms of transient response and sensitivity.

## 4. Conclusions

In this paper, an easy-to-fabricate and cost-efficient copper-tape-based, parallel-plate capacitive humidity sensor has been studied. The sensor is fabricated by using copper tape to make the plates of the capacitor, whereas tissue paper is used as a dielectric sensing layer. The sensor was tested in a homemade humidity chamber with a built-in DHT22 sensor, humidifier, dehumidifier, Arduino Mega 2560, and electric heater. Along with high performance in terms of response time and recovery time, sensitivity, repeatability, and stability, the use of such green sensors would certainly help reduce the e-waste that adversely affects our planet. Due to these advantages, such sensors can be used for various industrial and field applications.

## Figures and Tables

**Table 1:** Comparison of sensitivity, response time, and recovery time of different sensing materials with their sensing principles.

**Sensing Material**	**RH Range (%)**	**Sensitivity**	**Response Time**	**Recovery Time**	**Sensing Principle**	**References**
MWCNT/HEC	20–80	0.0485/%RH	11 s	35 s	Resistive	[45]
Paper	40–100	2 pF/%RH	250 s	175 s	Capacitive	[46]
PVA/dGO	40–100	68 kΩ/%RH	10 min	10 min	Resistive	[47]
CNT	20–80	-	6 s	120 s	Capacitive	[48]
GNP/CNF	30–90	140% (ΔR/R)	17 s	22 s	Resistive	[49]
PET	20–80	0.1%/%RH	-	-	Resistive	[50]
PI/Kapton	30–60	0.96%/%RH	-	-	Resistive	[51]
Smooth Paper	40–100	-	10 min	8 min	Impedance	[52]
Porous Paper	20–100	-	1 min	2–10 min	Capacitive	[52]
CAB/PET	20–80	1.2 pF/%RH	-	-	capacitive	[53]
Modified paper	10–95	-	25 s	188 s	Impedance	[54]
Tissue Paper	40–100	11 pF/%RH	1.5 s	2.2 s	Capacitive	This work

## Data Availability

Data underlying the results presented in this paper are not publicly available at this time but may be obtained from the authors upon reasonable request.
